# Cornerstone over Capstone: The case for structured career development opportunities early in the undergraduate biology curriculum as a way to influence science and biology identities

**DOI:** 10.1371/journal.pone.0285176

**Published:** 2023-05-05

**Authors:** Melissa McCartney, Jessica Colon

**Affiliations:** 1 Department of Biological Sciences, Florida International University, Miami, FL, United States of America; 2 STEM Transformation Institute, Florida International University, Miami, FL, United States of America; University of Texas Southwestern Medical Center at Dallas, UNITED STATES

## Abstract

Improving the rate at which individuals enter into STEM careers remains a national concern. STEM fields are currently facing a crisis with respect to filling jobs with qualified workers, suggesting that STEM jobs are available yet remain open and waiting for qualified graduates. Although researchers have previously investigated variables such as demographics and attrition rates for the lack of STEM graduates available to fill these job vacancies, there is a critical need for additional research examining the impact of additional career-related variables. To explore the impact of a biology-focused career development course (CDC), we surveyed 277 biology majors in their final semester who participated in the CDC. Respondents were asked to describe their perceptions of the professional development modules contained within the CDC and to describe what they would have done differently had the CDC been available earlier in their academic career. We grounded data analysis in science and biology identity frameworks. In agreement with earlier identity studies, we found that engagement with the CDC enhanced student’s performance/competence in biology and recognition as a biologist, two factors that are important for identity formation. Additionally, we show that students prefer to have the CDC earlier in their academic careers. Collectively, our data advance our understanding of career development of biology majors in two novel ways. First, we provide much needed qualitative data highlighting the mechanisms underlying the biology-focused CDC. Second, we provide both quantitative and qualitative data focused on the timing of the CDC, a topic which has not yet been purposely explored in biology.

## Introduction

For undergraduate STEM students who are discovering interests, exploring opportunities, and developing personal strengths, determining a future career path is challenging. For every student that has a clear career vision, there is a student struggling to identify a career path and another student changing a career goal [1]. Almost half of undergraduates in a STEM major choose to pursue different careers than originally intended, and many students continue to debate possible careers after their undergraduate education [2,3]. We should not assume that students are progressing toward intended STEM careers simply because they have persisted in STEM. In fact, only 44.6% of all degree holders in life sciences are employed in science and engineering occupations [4]. Although researchers have previously investigated variables such as demographics and attrition rates for the lack of STEM graduates available to fill job vacancies, there is a critical need for additional research examining the impact of career-related learning experiences during the undergraduate years [5–7]. It is possible STEM graduates do not enter the STEM workforce simply because they do not know how to navigate the options available to them.

One type of career-related learning experiences are career development courses (CDCs). CDCs are often provided as one-time, semester-long experiences and have been implemented and studied to better understand college students’ career decision-making skills [8]. Studies have shown that effects of CDCs were greater than those of individual counseling and other interventions [9–11]. In a review of CDC literature, Reardon et al., [8] confirm that the use of CDCs has strong, positive impacts on undergraduates career decision-making ability and other outcomes (*i*.*e*. the skills, knowledge, and attitudes acquired by participants, vocational identity, career maturity, and career decision-making). While CDCs have reliably been shown to increase student retention to graduation, their longitudinal impact on students entering the workforce remains unknown [8].

Very little research has been conducted regarding structured career planning within undergraduate STEM initiatives, *i*.*e*. STEM-focused CDCs [12]. The few studies that have been published on STEM-centered CDCs have yielded positive results, including increased student awareness and confidence toward STEM career preparation [1,13]. Other studies have focused on career interest as a predictor for STEM persistence and retention in the major [5,14] and how participation in a STEM-focused CDC decreased negative career thoughts, *i*.*e*. adverse consequences of student’s thinking related to career planning [12,15]. However, none of these studies provide qualitative data or a longitudinal context of their success.

It is critical for undergraduate educators to better understand the underlying potential of STEM-centered CDCs, especially in biology. A unique challenge for biology undergraduates attempting to enter the STEM workforce are students’ own career goals, which often include entry into highly competitive graduate programs in the health sciences [16]. However, fewer than half of medical school applicants (*e*.*g*., 42% in 2022 [17]) are accepted and enrolled in a U.S.-based medical school each year. These data suggest that over half of students (likely biology majors) who intend to enter medical school in the U.S. face a career barrier that forces them to adjust their career plans either late in their undergraduate careers or, most likely, after graduation when they no longer have access to university career services. Whether or not these biology graduates are retained in STEM fields post-career shift remains unknown [18].

The lack of a detailed understanding of STEM-centered CDCs, combined with the realization that a majority of biology majors will face a career roadblock, motivated us to develop a biology-specific CDC. Anecdotally, biology majors at our institution often see their education more as a checklist of courses to complete before applying to professional/graduate school and less as a comprehensive training to become a scientist, and, more specifically, a biologist. At its core, this is an identity crisis, and a first step forward may be to foster our students’ identity as a biologist rather than simply a future applicant to professional/graduate school. This is important, as science identity positively impacts the likelihood of entering a science occupation [19]. Therefore, we center our career development work in identity theories.

## Theoretical frameworks

### Science Identity

The construct of identity, or how individuals perceive themselves and engage others as a particular “kind of person” [20], has been widely studied across STEM in the context of student’s academic performance, engagement, career choice, and persistence in STEM [21–25]. To date, there is no firm consensus on the definition of science identity [26], however three major contributors are cited as necessary for science identity: 1) interest in the discipline, 2) recognition from others as a legitimate member of the discipline, and 3) performance and competence in the discipline [21]. *Interest* in STEM is a primary motivator of student’s STEM-related career choices as well as their identity development [27–29]. *Recognition* is an individual’s belief or perception that others, such as teachers, peers, and family members, consider them to be a STEM person [21]. *Performance/competence* is an individuals’ sense of both their ability to perform STEM tasks as well as understand STEM concepts [21,22].

Science identity is dynamic, and student trajectories may shift directions over time depending on students’ lived experiences and social interactions with others [30–33]. Identity begets identity, as students with a high science identity are more likely to make decisions that validate that identity [34]. Students with a high science identity may also be better able to maintain their motivation to persist in STEM fields because their efforts are directed towards a goal that aligns closely with their identity [35], while students with low science identity may withdraw in the face of increasing difficulty. Therefore, science identity may play a crucial role when students, and graduates, are deciding whether or not to remain in STEM when facing a roadblock.

### Disciplinary identity (biology identity)

Extending the more general science identity work into the more specific STEM discipline of physics, Hazari et al. [22] developed and tested a quantitative model for physics identity. This model is aligned with Carlone and Johnson’s [21] science identity framework of interest, recognition, and performance/competence within the discipline of physics. This model has since been validated in different postsecondary STEM disciplines, including biology and chemistry [18,36,37], mathematics [38], engineering [39], and computer science [40]. In more recent developments, sense of belonging has been added to the model as a fourth related construct, especially for senior undergraduates who have become immersed in disciplinary communities within their upper-level courses and departments [31].

A more granular understanding of identities across scientific disciplines, such as biology identity, chemistry identity, physics identity, *etc*., is critical for a comprehensive understanding of why differences in participation and engagement (*e*.*g*., gender differences) exist between disciplines [37,41]. This is relevant to career development in biology, as students’ career goals often necessitate entry into highly competitive graduate programs with acceptance rates of ~40%. It is highly likely that a majority of biology graduates will eventually face a career barrier, and a more comprehensive understanding of biology identity, specifically as it relates to biology careers, may help educators better prepare students to remain in biology if and/or when their career trajectories change. If we can better understand how to build career-related biology identity, we will be in a better position to scale career-related interventions to the extremely large populations of students who enter into undergraduate biology programs each year.

### Previous work and the current study

Previously, we designed and implemented a CDC composed of seven individual career development modules specific for biology majors. Participation in our CDC increased student’s science identity as well as student’s biology recognition and biology performance/competence [36]. While this previous work showed a quantitative, positive impact of the CDC, it did not provide an understanding of the mechanisms underlying the success of the CDC. Our current study expands on this previous work and fills two major gaps in the STEM-centered career development literature. First, the majority of research in this space is quantitative and does not capture students’ full experiences [42]. Therefore, there remains an untapped potential for more in depth, qualitative studies on student career-related learning experiences (*e*.*g*., CDCs) [43]. We respond by providing comprehensive, qualitative data explaining the mechanisms, *i*.*e*. the how and the why, underlying the influence of a biology-specific CDC on students’ science and biology identities.

Second, the timing of STEM-centered CDCs has not yet been purposely explored [44]. In this study, we only have one time point of science and biology identities taken when participants are at the end of their undergraduate career in biology. However, identity construction is fluid and can change from moment to moment and from context to context [30,44–47]. Because we are unable to go back in time and take earlier measurements of our participants identities, we decided instead to ask students when they would have preferred to have engaged with each module and, if they had participated earlier, how that would have altered their career development. We provide qualitative data underlying the benefits of providing a biology-specific CDC early in the curriculum as a way to provide students more guidance in organizing their undergraduate experience to better prepare them to enter their desired career fields.

Specifically, the research questions guiding this study were:

1. What are the mechanisms underlying the shift in student’s science and biology identities while participating in a biology-specific CDC?
2. What is the optimal timing for a biology-specific CDC?

## Methods

### Student demographics

Florida International University **(**FIU) is a public Carnegie R1-ranked urban university and Hispanic Serving Institution (HSI) enrolling 48,664 undergraduates (Fall 2021), of which 63.9% are Hispanic, 12.5% are Black or African American, 10.2% are white, 2.64% are Asian, 2.18% are two or more races, less than 1% are native Hawaiian or other Pacific Islanders, less than 1% are Native Americans, and 57% are women. The biology major had 4,159 students enrolled, composed mostly of Hispanic students and large numbers of First Generation (~20%) and Pell-eligible students (~51%). The demographics of students in this study mirror overall FIU demographics. Our student population, a portion of which provided data, is 82% Hispanic, 9% Black or African American, 4% white, 2% Asian, and 3% reported other/unknown. Our student population is 71% women, 23% First Generation, and 57% Pell eligible (Table 1).

**Table 1:** An overview of the student population who participated in the CDC over two semesters. Data shown represent students enrolled in the study, a subset of which chose to complete the questionnaires.

	FIU	study population
number of students	48,664	277
Hispanic	63.9%	82%
Black of African American	12.5%	9%
White	10.2%	4%
Asian	2.6%	2%
two or more races	2.2%	none reporting
Native Hawaiian or Pacific Islander	less than 1%	none reporting
Native Americans	less than 1%	none reporting
women	57%	71%
first generation	20%	23%
Pell eligible	51%	57%

### The CDC: Senior Seminar

Senior Seminar (BSC 4931) is a one-credit Capstone course required for graduation with a biology degree. Participants in this study enrolled in Senior Seminar during the Fall 2020 and Spring 2021 semesters with no knowledge of the curriculum for their chosen section. Both semesters were during the COVID pandemic with the course being taught synchronously and remotely using Zoom software.

For this study, sixteen different sections of Senior Seminar were taught by six different instructors over two semesters using the 7-module CDC curriculum (Fall 2020: 8 sections, 4 instructors, 144 students; Spring 2021: 8 sections, 5 instructors, 133 students). To standardize instruction across sections and semesters, instructors involved in this study met weekly to review upcoming lectures and to debrief on completed lectures. Previous data analysis revealed no significant differences between instructors, suggesting that any slight differences among instructor teaching style had no substantive effect [36]. The seven career development modules contained within the CDC are described in Appendix 1 in S1 File.

### Data collection

This study was deemed exempt by the Florida International University Institutional Review board (FIU IRB) (IRB-20-0015-AM01). Participants consisted of students enrolled in a participating Senior Seminar section (Table 1) and responded to the questionnaire the same semester in which they were enrolled. Written consent, using a consent form approved by the FIU IRB, was collected from all students who completed the questionnaire and no data from minors were collected. Students were given course credit for completing the questionnaire regardless of whether they agreed to have their data included and the questionnaire was not associated with any graded activities. We collected open-ended responses to the questions “please explain why the [module] did, or did not, make you feel more like a scientist” (asked during Fall 2020) and, in reference to when students would have preferred to engage with the modules, “how would having the [module] during your chosen time have better prepared you for life after FIU? What would you have done differently in regards to career preparation?” (asked during Spring 2021). Each open-ended question was asked separately for each module, for a total of 7 open-ended questions each semester. In the Fall of 2020, we had an average of 117 students responding with the average length of responses being 23 words (~81% response rate). In the Spring of 2021, we had an average of 98 students responding with the average length of responses being 28 words (~74% response rate). Complete response information is shown in Appendix 2 in S1 File. The data that support the findings of this study are available from the corresponding author, [M.M], upon request.

We asked one quantitative question: “At what stage of your college career would the [module] discussion have been most helpful?” This was a single-answer multiple choice question with answer selections of: 1) freshman year, 2) sophomore year, 3) junior year, and 4) senior year.

### Qualitative data analysis

Student open-ended responses were coded using both deductive and inductive coding techniques in order to both target specific constructs (science identity) while still leaving room for discovery (biology identity and timing of CDC implementation) [48]. Deductive coding is a top down approach where predetermined codes are used to analyze data. In our case, we used specific questionnaire items that we used to quantitatively measure science identity previously [36,49] (Appendix 3 in S1 File). Our qualitative analysis was completed without knowledge or reference to students’ responses to the quantitative questionnaire items. The science identity items (deductive codes) are written in a manner that is similar to how our students would talk/write, and we were able to see direct connections to “I have a strong sense of belonging to the community of scientists,” “I have come to think of myself as a scientist,” and “I feel like I belong in the field of science.” Any open-ended response that connected to one of the deductive codes was coded.

We also performed inductive coding, a subset of thematic analysis [50]. Per definition, inductive coding is free from theoretical frameworks. Instead, inductive coding is completely driven by the participants’ responses [50]. Inductive coding was used to identify interesting codes that emerged from the open-ended responses (related to biology identity and the timing of CDC implementation). While we know that biology identity is composed of identity, recognition, and performance/competence, it was likely that student answers were general in nature and looking for emerging themes related to disciplinary identity would provide a more granular understanding of student open-ended responses. In addition, a majority of the biology identity items are not written in the manner that our students would generally talk/write themselves. For example, it is unlikely that a student would write “my biology teacher sees me as a biology person” in an open ended response. Therefore, we opted for inductive coding and then grouped the resulting codes under the three factors of biology identity.

For inductive coding, three researchers read all of the open-ended responses and independently created inductively generated lists of the different perceptions, attitudes, and opinions that arose from participant responses. Initial findings were discussed among the three researchers and a preliminary code book was developed consisting of short, descriptive phrases that could be used to describe particular perceptions, attitudes, or opinions expressed by participants. Using this team-generated code book, each open-ended question was then independently coded by two researchers. All our codes are presented in either data figures or in Appendices 4 and 5 in S1 File. All researchers then convened to discuss, further define, and reduce codes that were unclear. Analysis of coding considered only the presence or absence of specific codes within each open-ended response, not the frequency with which a single participant expressed a particular code. Responses corresponding to more than one code were coded to each code they corresponded with. Kappa values measuring inter-rater reliability (the extent to which researchers assign the same code to the same data) were over 0.85, which represent higher standards than recommended (0.65) [51]. All qualitative analysis was completed using Nvivo software (NVivo version 12, QSR International). Once all the inductive coding was completed, we examined how/whether the codes aligned to our disciplinary identity framework.

## Results

### Mechanisms underlying science identity

**Fig 1:**
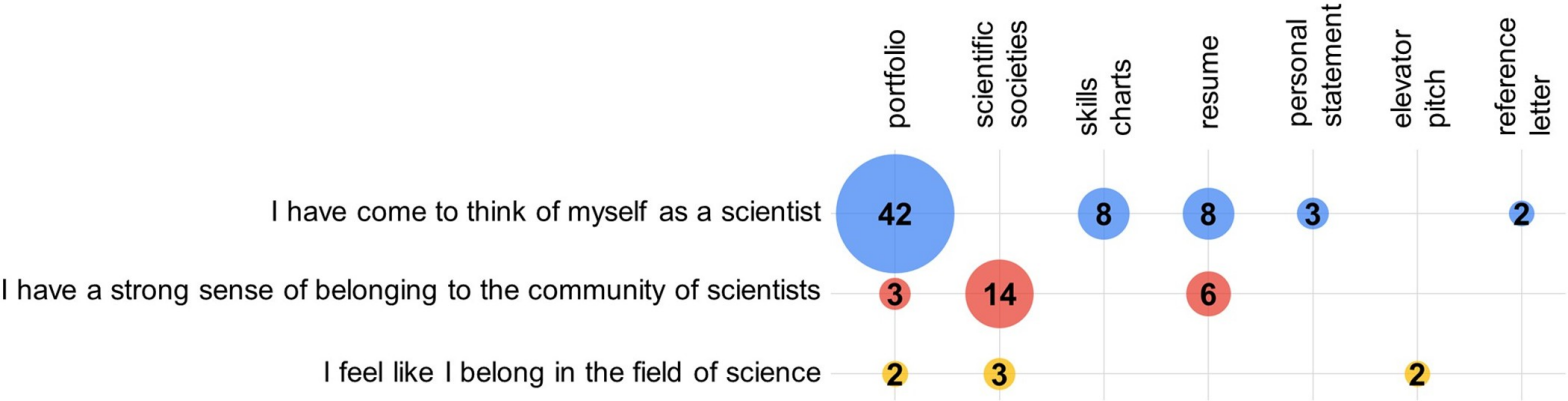
Deductive coding of student short answer responses to the question “please explain why the [module] did, or did not, make you feel more like a scientist” using the Estrada (2011) science identity questionnaire as codes (Appendix 3 in S1 File). The y-axis shows three codes that were found in the data set and the x-axis indicates which module the students connected to each code. The data are shown graphically, with the area of each circle being representative of the percentage of students including each science identity item in their response (the exact percentage is noted within each circle). *n* values for student responses range from 115–118 and are described in Appendix 2 in S1 File.

An overview of results from deductive coding are shown in Fig 1. Each module was found to have a connection to science identity, with portfolio, scientific societies, and resume modules connecting to more than one code, even if some of these connections represented a small number of students. The deductive code “I have come to think of myself as a scientist,” *i*.*e*. the definition of science identity, spanned across open-ended responses for five of the modules. We see 42% of students connect “thinking of themselves as a scientist” to the portfolio module.

A closer look at student open ended responses from the portfolio module connecting to “I have come to think of myself as a scientist” centered on students now being able to place the entirety of their undergraduate experience into the new context of being scientists, in whichever way they are personally defining what a scientist is (Table 2). This code also emerged for the skills charts (8%), resume (8%), personal statement (3%) and reference letter (2%) (Fig 1). Similar to the portfolio responses, students mention being able to place the entirety of their undergraduate experience into the new context of being scientists (Table 2).

**Table 2:** Representative open-ended responses to the prompt “please explain why the [module] did, or did not, make you feel more like a scientist” that connect to the deductive code of “I have come to think of myself as a scientist”. Relevant sections of the student response are bolded and underlined.

**module**	Representative open-ended responses to the prompt “please explain why the [module] did, or did not, make you feel more like a scientist” that connect to the deductive code of “I have come to think of myself as a scientist.”	
**scientific** **portfolios** (42% of responses)	The portfolio presentation did make me feel more like a scientist because it helped me remember all the biological activities I have done over the years and **was able to show my abilities as a scientist.**	The portfolio presentation made me dig and **realize the skills that I had acquired as a student and scientist** throughout my collegiate experience. **Skills that I had not considered to make me a scientist** due to always focusing on what I am lacking instead of what I have learned.
**skills** **chart** (8% of responses)	This job search made us **validate the skills we already posses** and more accurately create plans to acquire those we lack. This too **validated the skills I had acquired in my lectures and lab that I did not realize made me feel like a scientist.**	I was directed into very scientific fields. **It made me feel capable based on my values, thoughts and work ethics that I could be involved in the field**.
**resume** (8% of responses)	It helped me understand how much I can **relate my experience as a pre-med student to being a scientist** and having a huge amount of experience in labs, and scientific writing.	The CV and resume writing workshop made me feel more like a scientist because **I found out I actually have many more science-related skills than I previously thought I did.** Getting help from the professor as to what I can add to my resume, in the end **I felt more like a science person when I looked at my completed resume and past experience**.
**personal statement** (3% of responses)	It made me analyze and realize **how much I am involved in the biological sciences.**	The personal statement allowed me to feel as I was **confident in my scientific ability**.
**reference letter** **(**2% of responses)	Writing down what others would think of me, especially professors, **assisted me to further realize that I am a scientist.**	Writing my own recommendation letter allowed me to **think of my achievements and attributes I have to offer.** Given that a rec letter comes from a science professor usually if your major is in the sciences **it does make me feel more like a scientist.**

### Mechanisms underlying biology identity

**Fig 2:**
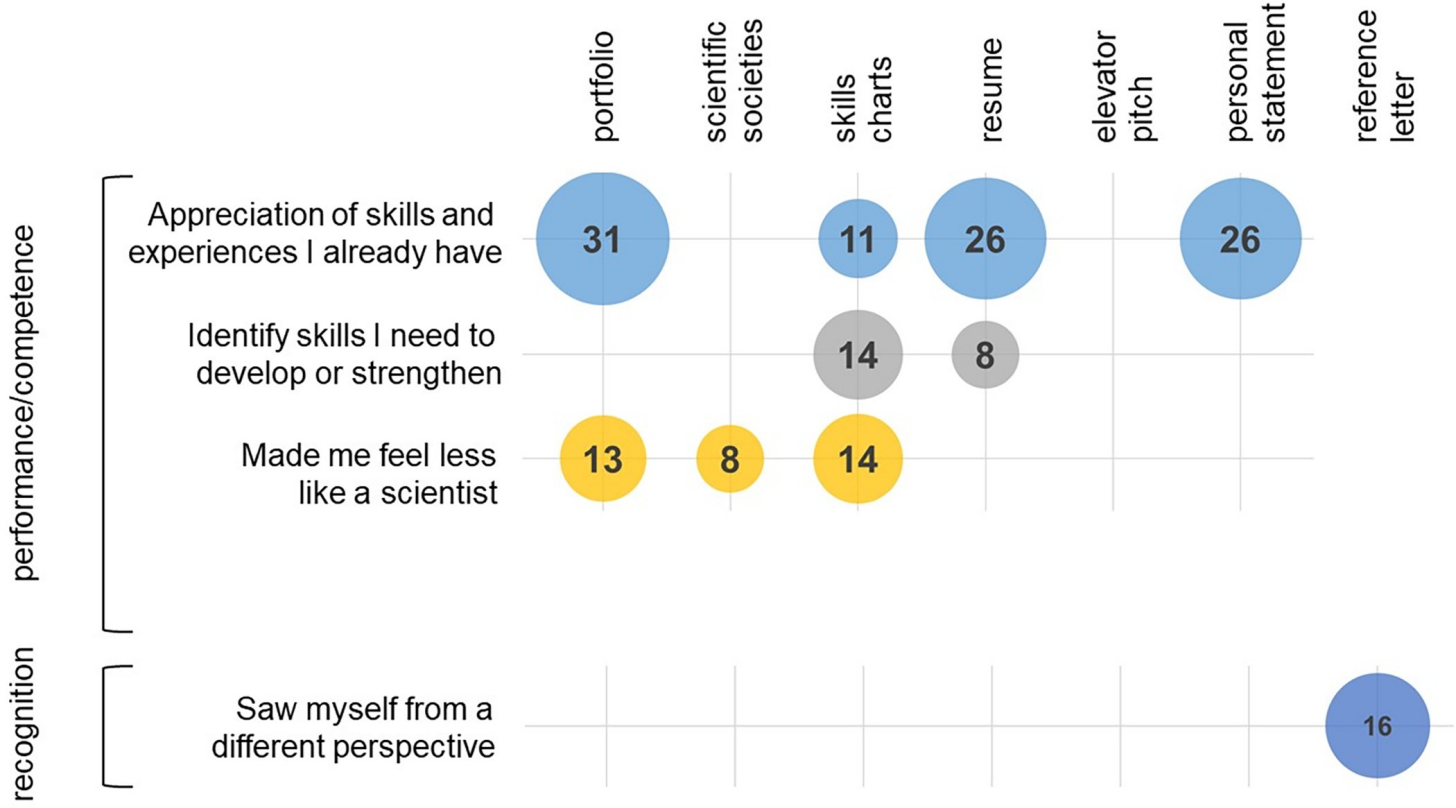
Emerging (inductive) codes that connect to biology identity. The y-axis shows codes connecting to constructs of biology identity that were found in the data set and the x-axis indicates which module the students connected to each code. The data are shown graphically, with the area of each circle being representative of the percentage of student responses connecting to each code (the exact percentage is noted within each circle). *n* values for student responses range from 115–118 and are described in Appendix 2 in S1 File.

Deductive coding was appropriate for exploring science identity for two reasons: 1) only one factor is measured, which is science identity, and 2) science identity items are written in a manner that is similar to how our students would talk/write, which enabled us to see direct connections to three separate deductive codes. In contrast, biology identity is more complex, is composed of three factors, and items are not written in a manner that is similar to how our students would talk/write. Therefore, we opted for inductive coding in order to develop a more granular understanding of the data. The deductive science identity coding and the inductive biology identity coding are not meant to be directly compared to each other as each identity framework has its own unique characteristics. We present the emerging codes that are most connected to biology identity (Fig 2) (a complete list of codes can be found in Appendix 4 in S1 File). We grounded interpretation of these codes in a biology identity framework consisting of performance/competence, recognition, interest, and sense of belonging [21,22,31].

### Connections to performance/competence

The majority of codes we encountered for students biology identity connected to performance/competence, specifically “appreciation of skills and experiences I already have” (Fig 2). This connection occurred across four separate modules (portfolio 31%, skills charts 11%, resume 26%, and reference letter 26%), suggesting that a common, uniting theme among these modules is their ability to engage students in reflection on the skills and experiences they may not have previously connected to their ability to understand biology and to perform in biology. Example responses from students are shown in Table 3.

**Table 3:** Representative open-ended responses to the prompt “please explain why the [module] did, or did not, make you feel more like a scientist” that connect to the example student responses connecting to the inductive code “appreciation of the skills and experiences I already have”. Relevant sections of the student response are bolded and underlined.

**module**	Representative open-ended responses to the prompt “please explain why the [module] did, or did not, make you feel more like a scientist” that connect to the example student responses connecting to the inductive code “appreciation of the skills and experiences I already have.”		
**scientific portfolios** (31% of responses)	It…made me realize I have a lot of experience of…a scientists. we as stem majors have so many hours of laboratory experience that has prepared us to be scientists in the field we desire to pursue. This portfolio helped me **gather all my experiences and realize the amount of science knowledge I’ve had over my collegiate years**.	I have taken numerous courses in the field of biology and the portfolio allowed me to gather all of that information and put it into a single presentation. **The amount of hours in class and lab really show once you compile it and explain what you have learned in these classes**.	It allowed me to see all that I have done during my time at FIU and see that **I’ve actually done a lot more than I thought. It helped me be able to quantify how much I’ve done.**
**skills charts** (11% of responses)	It showed me that **I can already hold a more science related role** in based off of my current experience.	The job searches helped me find more opportunities in the things I have yet to accomplish for my future endeavors. The jobs also **showed me how qualified I am in certain areas.**	The job searches made me feel like a scientist because I gained information on what the requirements were to apply for those jobs. And **I also saw that qualities I already had for the job**.
**resume** (26% of responses)	The CV and resume made me feel more of a scientist because it helped me **realize how much of the things i do are science based.**	The CV and resume workshop did make me feel more like a scientist for similar reasons as the presentations- it **made me look through and expand on my experiences that I initially did not think were "enough ".**	The resume allowed me to demonstrate my skills and assets to any time. It **allowed me to realize my achievements as a scientist**.
**reference letter** (26% of responses)	It helped me **reflect on myself as a student and as a professional**	Writing my own rec letter **allowed me to think of my achievements and attributes I have to offer**. Given that a rec letter comes from a science professor usually if your major is in the sciences it does make me feel more like a scientist.	Writing my own rec letter made me feel more like a scientist because I was able to **present my skills and qualities as a good candidate for graduate school**. A nice opportunity to remember myself that I am valuable and I have accomplished many goals.

Collectively, across modules, student responses describe how the module allowed them to see their scientific skills and experiences as a whole entity rather than as smaller parts of several individual courses (Table 3). This connects to the other common topic across student responses, which is being able to place the skills and experiences gained during their undergraduate education into a new context of being a scientist, being a biologist, or the world outside of their undergraduate classroom/institution in general.

We also see student responses connecting to performance/competence through a somewhat opposite lens, specifically identifying skills and experiences that *still need* strengthening (Fig 2, Table 4). Interestingly, these comments are not presented in a negative way. Instead, student responses are placed in the context of being pleased with what they have already learned and experienced yet striving to learn and experience even more. We elected to keep these responses within the context of performance/competence as they describe student’s ongoing pursuit to further understand biology and to better perform in biology.

**Table 4:** Representative open-ended responses to the prompt “please explain why the [module] did, or did not, make you feel more like a scientist” that connect to the inductive code “identify skills I need to develop or strengthen”. Relevant sections of the student response are bolded and underlined. These open-ended responses are representative of the code “identify skills I need to develop or strengthen”.

**module**	Representative open-ended responses to the prompt “please explain why the [module] did, or did not, make you feel more like a scientist” that connect to the inductive code “identify skills I need to develop or strengthen.”		
**skills charts** (14% of responses)	They help me identify which skills I already know and have but **can also tell me skills that I can still learn to become a better scientist.**	This job search made us validate the skills we already posses and **more accurately create plans to acquire those we lack**. This too validated the skills I had acquired in my lectures and lab that I did not realize made me feel like a scientist.	Job searches made me realize **I need more skills and experiences** in order to be a more suitable candidate.
**resume** (8% of responses)	It helped put all the experience we’ve gained down on a piece of paper and see how much has been done and **what else needs to be done.**	**There is still a lot needed to be done.** The CV only somewhat pointed this more out to me. It did help me know how to write one though which was great. It also helped a lot what to avoid or add in my resumes.	Writing my CV **helped me realize where I needed to improve** and strengthen my resume in order to be more competitive in my field of work.

We see student responses indicating that they felt *less* like a scientist after participating in the CDC (Fig 2, Table 5). We include these student responses in the performance/competence category as a cautionary warning that performance/competence can be influenced in several ways. While making students feel less like scientists was not our intent, these data are significant (portfolio 13%, scientific societies 8%, and skills charts 14%) and need to be considered. Some responses in Table 5 mention feeling inferior to peers, which is to be expected among biology majors, the majority of whom are on a competitive pre-health career path.

**Table 5:** Representative open-ended responses to the prompt “please explain why the [module] did, or did not, make you feel more like a scientist” that connect to the inductive code “made me feel less like a scientist” Relevant sections of the student response are bolded and underlined.

**modules**	Representative open-ended responses to the prompt “please explain why the [module] did, or did not, make you feel more like a scientist” that connect to the inductive code “made me feel less like a scientist”		
**scientific portfolios** (13% of responses)	The portfolio made me feel less of a scientist because **I only had work that was non-science related in my file.**	I’ve come to realize that **my background in science is not as strong as other students in my field**, which makes me feel insecure as a biology pre-med student.	I don’t think science is a strength of mine and doing the portfolio really emphasized that feeling to me, **I realized I really lacked experience and that I also lack passion for the field.**
**scientific societies** (8% of responses)	The scientific societies made me feel less of a scientist because it **makes you feel like you aren’t doing or aren’t apart of enough societies.**	They made me feel less like a scientist since **I was not a part of any of these societies.**	**It kind of intimidated me**, because I had gone through all of undergrad not realizing this was a thing.
**skills charts** (14% of responses)	For many of the positions, **experience was required. I unfortunately did not have any**, but though I did not feel like a scientist it inspired me to get more involved and to not quit looking for a job in the field.	The job search did not make me feel like a scientist. **It made me feel like a worker looking for work** because I need money to pay my bills.	It did not make me feel like a scientist, **because for most of the job searches I required further graduate studies**.

### Connections to recognition

Recognition is one’s perception of how others view them in relation to the subject (biology) [31]. We see only one set of student responses that connect to how they are viewed by others, specifically with the reference letter module (Fig 2). For example:

*This **helped me think from a professor’s perspective** and also how to write my experiences and accomplishments from their point of view*.

*It just functioned more so as an evaluation of **who you are to the eyes of another individual** and not so as something that necessarily defined yourself as a scientist because depending on who the letter is from, you yourself can be seen in completely different perspective. Although it is to note that this in the context of a Biology student so many instances these letters will be evaluated from a scientific lens so this distinction can be seen as appropriate*.

*Writing my own recommendation letter was a little be weird since **I had to think I was a professor recommending someone**. As a scientist I was able to incorporate thinking abilities to do it*

### Connection to interest

We saw no student open-ended answer responses connecting to their interest in biology. Previously, we saw no change in student interest in biology using quantitative assessments [36], consistent with the lack of mention of interest in biology in student qualitative responses. This is in agreement with previous research suggesting that interest becomes a less important identity construct as students progress through the major since they are all interested enough to be majoring in biology [21,31].

### Connection to sense of belonging

We see a connection to sense of belonging, the sometimes fourth construct of biology identity, in the science identity coding data presented in Fig 1. While these data were analyzed with science identity as a framework, their connection to sense of belonging also places them within biology identity frameworks. Specifically, the codes “I have a strong sense of belonging to the community of scientists” and “I feel like I belong in the field of science” emerged for modules that were impacting students’ sense of belonging (Fig 1). The scientific societies module was most connected to sense of belonging, with 14% of responses connecting to “I have a strong sense of belonging to the community of scientists” and 3% of responses connecting to “I feel like I belong in the field of science” (Fig 1). Collectively, student responses centered on realizing, likely for the first time, that they are eligible to join scientific societies (Table 6). Related to the portfolio and resume modules, we found connections to students realizing they already are active in the scientific community. With the elevator pitch module, we see students describing gaining confidence in themselves and realizing that they are a part of the scientific community.

**Table 6:** Representative open-ended responses to the prompt “please explain why the [module] did, or did not, make you feel more like a scientist” that connect to the deductive codes of “I have a strong sense of belonging to the community of scientists” and “I feel like I belong in the field of science”. Relevant sections of the student response are bolded and underlined.

**modules**	Representative open-ended responses to the prompt “please explain why the [module] did, or did not, make you feel more like a scientist” that connect to the deductive codes of “I have a strong sense of belonging to the community of scientists” and “I feel like I belong in the field of science.”	
**scientific portfolios** (3% / 2% of responses)	While putting my portfolio together I realized that **I have been very active in the science community.**	
**scientific societies** (14% / 3% of responses)	It helped me realized **that I belong to this community of scientists.**	The scientific societies allowed me to join communities that are interested in the same things I am, **gives me a sense of belongingness within the scientific fields**.
**resume** (6% / 0% of responses)	I had been procrastinating to build my CV so this was the perfect opportunity to do it. As I was listing my achievements and experiences **I felt as part of the scientific community**.	I somewhat felt like a scientist as **some of the groups i was involved with were science related**
**elevator** **pitch** (0% / 2% of responses)	It boosted up my confidence and made **me feel like I can be a part of the scientific community**	The elevator pitches were extremely helpful in not only forcing me to come out of my comfort zone (by the fourth class I was fully comfortable with speaking!) but also made me find the key traits about myself **that made me a valuable member to the scientific society** and elaborate on them in a quick and concise way.

### Additional inductive codes connecting to “please explain why the [module] did, or did not, make you feel more like a scientist”

A complete list of additional, inductive codes that were not related to science or biology identity found in these data are shown in Appendix 4 in S1 File. Overall, the majority of these additional, inductive codes relate to specific logistics of each module, *e*.*g*. organization of experiences, how to write a personal statement, or how to ask for a reference letter. These data are relevant when implementing modules and provide insight into additional benefits of the modules for students.

### Student’s preferred timing of module implementation

**Fig 3:**
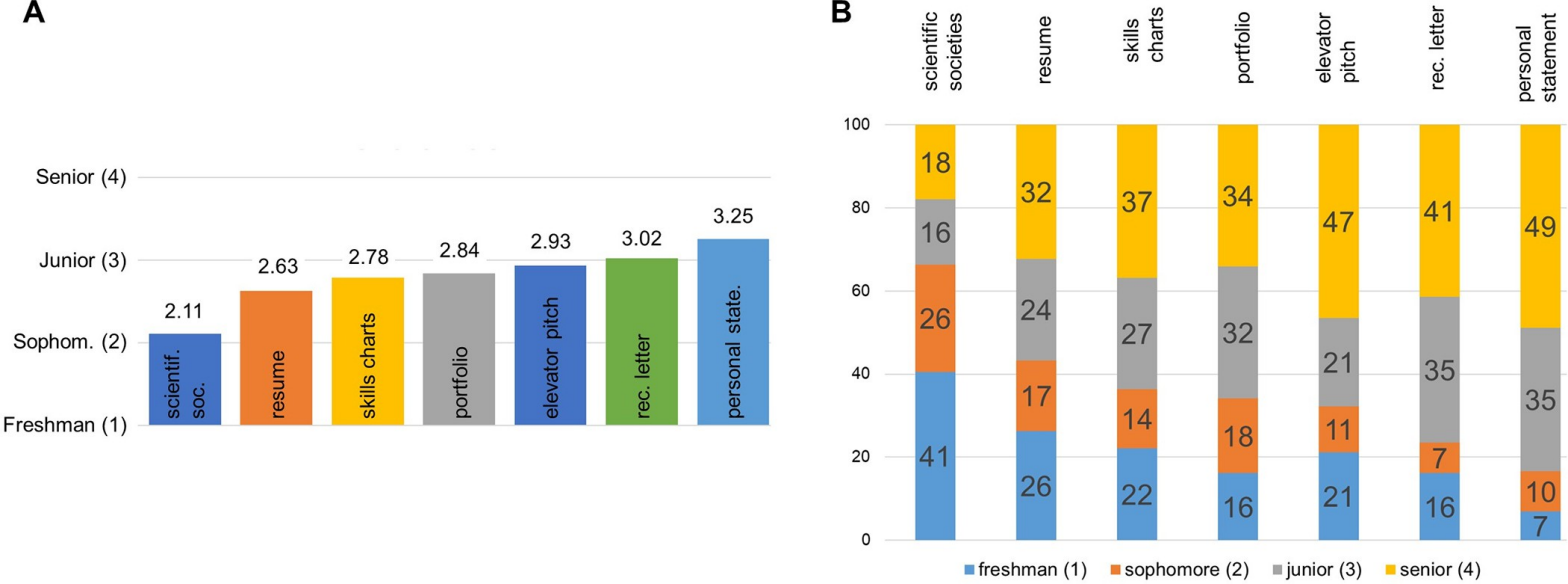
When students would have preferred to engage with each module. We asked one quantitative question: “At what stage of your college career would the [module] discussion have been most helpful?” This was a single-answer multiple choice question with answer selections of 1) freshman year, 2) sophomore year, 3) junior year, and 4) senior year. Panel A shows the average response for each module (*n* = 217). Panel B shows the distribution of each choice (freshman, sophomore, junior, or senior) by percentage of student responses.

We asked students (quantitatively) when they would have preferred to have each module (Fig 3). The preferred timing for these modules is between students’ sophomore and junior years.

**Fig 4:**
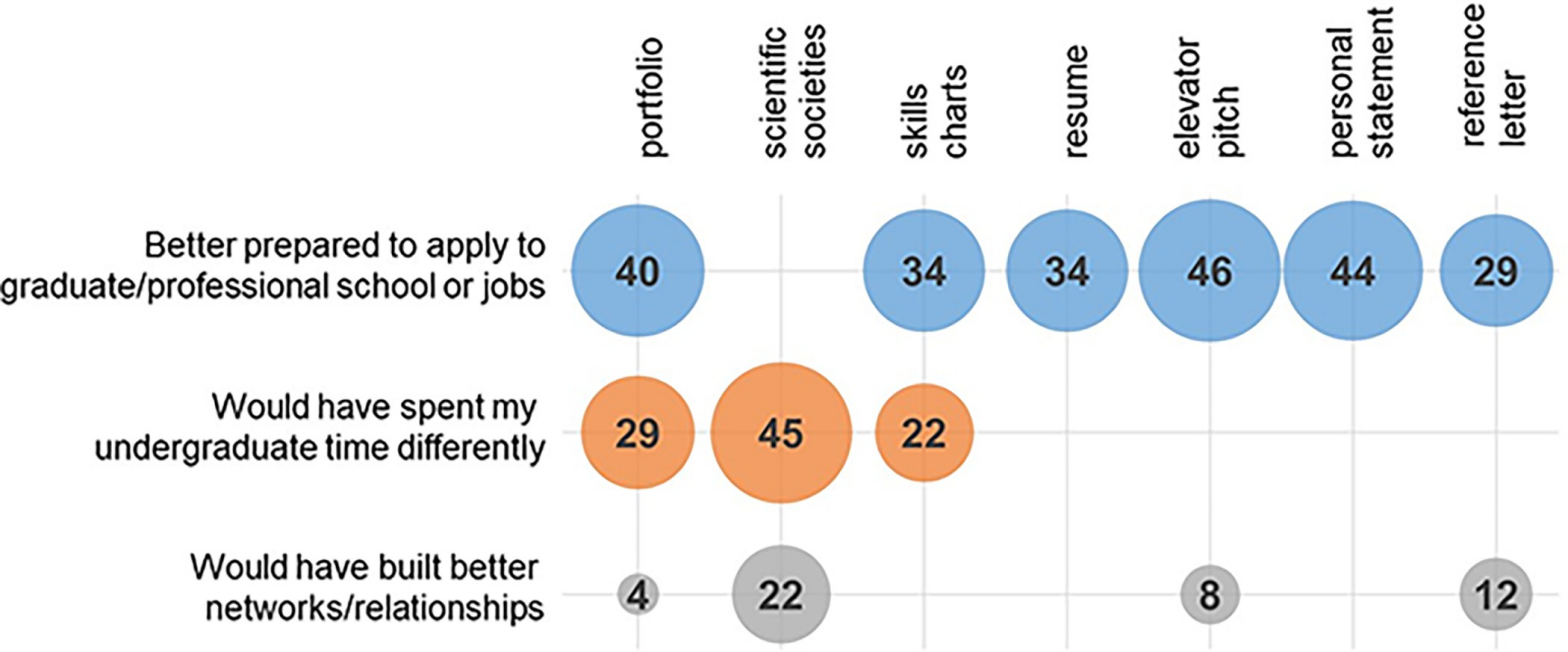
How having modules earlier would have benefitted students. The y-axis shows inductive codes connected to student responses that were found in the data set and the x-axis indicates which module the students connected to each code. The data are shown graphically, with the area of each circle being representative of the percentage of student responses connecting to each code (the exact percentage is noted within each circle). *n* values for student responses range from 97–99 and are described in Appendix 2 in S1 File.

To explore these data qualitatively, we asked students “How would having this assignment/information during your chosen time have better prepared you for life after FIU? What would you have done differently in regards to career preparation?” for each module. Here, we present the three most relevant codes that are likely to influence science identity (Fig 4) (a complete list of codes are found in Appendix 5 in S1 File).

### An earlier implementation could lead to students being better prepared to apply to post-grad opportunities

For almost every module, we see students stating that engaging with these modules earlier in their career would have allowed them to be more prepared to enter post-grad life (Fig 4). Most often, students detail how knowing how to prepare professional documents and present themselves earlier would have made it easier to find internships and opportunities on campus (Table 7).

**Table 7:** Representative open-ended responses to the prompt “how would having the [module] during your chosen time have better prepared you for life after FIU? What would you have done differently in regards to career preparation?” that connect to the inductive code of “better prepared to apply to graduate/professional schools or jobs” Relevant sections of the student response are bolded and underlined.

**modules**	Representative open-ended responses to the prompt “how would having the [module] during your chosen time have better prepared you for life after FIU? What would you have done differently in regards to career preparation?” that connect to the inductive code of “better prepared to apply to graduate/professional schools or jobs”		
**scientific** **portfolios** (40% of responses)	In that point in my life, I was applying for multiple REU programs. This would have **allowed me to have a more clear idea towards how I had to format my portfolio and what should be included**.	Well not for life after FIU but for life during FIU. I took jobs during my sophomore year that required interviews, and i think **being more prepared for those would have landed me the jobs I desired instead of the jobs I had to settle for**.	It would allow more time to prepare for what’s to come after graduating. Would allow more time to grow your resume with experience and **think about career choices before graduation and grad school apps.**
**resume** (34% of responses)	Learning how to create a strong resume during sophomore year **may have landed me better internships to apply for in the next upcoming years**. I started apply for internships and jobs during my junior and senior year so this skill would have benefited me more during sophomore year	Freshman year is the best year to get a resume because students can start preparing for life after FIU early. Many of us also work while going to college. **Having a good resume helps to get good jobs and even internships that could prepare us for life after FIU**.	This would have benefited me earlier on as **I did start to show my resume to jobs and research places early on and I learned a lot later that I did not have correct formatting or information that I should have** and I can’t imagine how unprofessional or unexperienced that could have made me look. This would help so many incoming students.
**elevator** **pitch** (46% of responses)	It would have given me a **greater understanding of how small talks with relevant people in the my desired field of study and work are important for any job position** I was interested in seeking.	I feel i could have used this **just a bit earlier to get into research labs and "pitch myself" to professors**	during the sophomore year is when I started to look for opportunities outside of school so **it would have been good to know how to approach someone that could offer me a job or volunteer position**.
**personal** **statement** (44% of responses)	Junior year is when I looked into different internships and noticed some would ask for personal statement where I had no clue what it was and **ended up not applying because of this. If I was introduced to personal statements sooner I may have applied for internships** that require this.	**I had no idea what a personal statement was until I took senior seminar.** I believe this would be helpful for any student, regardless of their academic standing.	Personal statements are extremely important and a contributing factor for many post- undergraduate programs and schools. A personal statement workshop during junior year would **have helped me not only to make a better personal statement, but even to have been able to apply to medical school earlier on.**
**reference** **letter** (29% of responses)	**Throughout undergrad, there are many summer programs/ jobs/ internships that will ask for letters of recommendation** so I believe it would have been better to learn this early on.	It would have been useful since **it would have helped me to better prepare my application for a better-paid job.** Having/knowing how to write my own recommendation letter during freshman year was a good template to further prepare and ask to professor for letters for professional school, internships applications, scholarships	I might **have been able to apply to graduate school earlier** if I would have had this opportunity

We see a slightly different trend in responses for the skills chart module (34% of responses). Specifically, many students describe how learning how to better connect their Biological skills set to available jobs would have directly benefited them:

*Would have **seeked out jobs that would benefit my career** not just pay the bills*.

*I think sophomore year **since my program requires direct patient care, I was actually looking for a job** my sophomore year*.

*Some students need to enter the workforce after high school. This activity during freshman year **would have been an encouragement for finding a better-paid job**, and also emphasize my need for improvements in the skills I am/was lacking*.

We also learned about one success story as a direct result of this module:

*I am considering a gap year and was planning to switch from my current job position, I actually applied to the job positions I wrote in these charts and got a job- this assignment was great!*

### An earlier implementation could lead to a better structured undergraduate experience

Students describe how earlier engagement with the modules would have enabled them to spend their undergraduate years more efficiently (Fig 4). For the portfolio module (29% of responses), we see students describing how they would better frame their college experience, specifically with getting more involved in career-related opportunities (Table 8). We see similar responses for scientific societies (45%). Specifically, students describe how awareness of the existence of scientific societies and clubs early on in their academic careers would have led to them becoming more involved earlier, when the benefits of membership could have helped in their career development (Table 8). We see a different subset of responses for skills charts (22% of responses). Most often, responses focused on how students were able to realize that you can do more with a biology degree than go to medical school and wished they had learned this earlier (Table 8).

**Table 8:** Representative open-ended responses to the prompt “how would having the [module] during your chosen time have better prepared you for life after FIU? What would you have done differently in regards to career preparation?” that connect to the inductive code of “would have spent my undergraduate time differently.” Relevant sections of the student response are bolded and underlined.

**modules**	Representative open-ended responses to the prompt “how would having the [module] during your chosen time have better prepared you for life after FIU? What would you have done differently in regards to career preparation?” that connect to the inductive code of “would have spent my undergraduate time differently”		
**scientific portfolios** (29% of responses)	**It would have pushed me more to join clubs and branch out** in order to be able to add more to my portfolio instead of rushing it all at the end.	I would have planned way ahead of time for my future aspirations as a medical doctor by **getting more involved in medical clubs and meeting medical professionals**	It would have let me understand better the different types of STEM majors there are out there. **I would have probably joined more STEM related club in order to prepare myself in a career.**
**scientific societies** (45% of responses)	**Had a joined some of the premedical societies that I found in this assignment earlier then I would have had better mentorship and guidance towards my future career**. I think this assignment would be worthwhile to put in freshman year SLS so student get involved with their future fields early on.	**Looking for scientific societies early on would have provided me with a myriad of opportunities that I had no idea even existed.** For a pre-med student seeking to attend medical school, seeking scientific societies as a senior holds little value. **These scientific societies would have allowed me as a freshman to build up networks, participate in numerous activities, volunteer opportunities, perhaps shadowing or research.** All of these are very hard to encounter on your own and would have been greatly facilitated by the activity of seeking out scientific societies given in senior seminar.	I think it would have been most helpful to introduce these freshman year in order to benefit as much as possible from membership in these societies. **Being involved early in all these societies could have provided involvement, leadership opportunities, resume building and networking that all are great to develop early in your undergraduate career.**
**skills charts** (22% of responses)	it made me aware of all of the job opportunities available to students with a B.S. **It also brought to my attention how biology-related jobs are not solely limited to medical/dental/pharmacy school and teaching.** Other positions exist in the world, though not as common.	**My freshman mind could have broadened earlier on,** rather than just believing that the Biology major was for doctors.	Have a better understanding as to what I want in my future. **It would have prepared me to better understand that there are many opportunities I can do with my degree.**

### Building better networks

Across four modules, we see students describing how they would have spent more time networking, and built stronger networks, if they had engaged with the modules earlier (Fig 4). This suggests that implementing these modules earlier may influence students recognition in biology, as networking would have allowed for more of their peers, colleagues, instructors, and professors to see them as biologists (Table 9). Where we see a slight difference in student responses is with the reference letter module (12% of participants). Previously, student responses described general networking, but here we see specific references to developing relationships with professors (Table 9).

**Table 9:** Representative open-ended responses to the prompt “how would having the [module] during your chosen time have better prepared you for life after FIU? What would you have done differently in regards to career preparation?” that connect to the inductive code of “I would have built better networks/relationships.” Relevant sections of the student response are bolded and underlined.

**modules**	Representative open-ended responses to the prompt “how would having the [module] during your chosen time have better prepared you for life after FIU? What would you have done differently in regards to career preparation?” that connect to the inductive code of “I would have built better networks/relationships”		
**scientific portfolios** (4% of responses)	I would have **made a larger effort to put myself out there and create network with others in my career**	As a sophomore, being given this assignment/information would’ve definitely better prepared me for life after FIU. I think **I would’ve done more for my network; put myself out there.**	**I began to work a lot and make connections.** Information last year would have showed me how to better manage time and stay professional.
**scientific societies** (22% of responses)	As a freshman, being exposed to scientific societies **would’ve definitely had me interacting and making relationships with people** which is important for networking purposes.	Knowing more about scientific societies earlier **could have helped me improve my networking skills earlier in my college career**	Being exposed to different societies during freshman year would have been a better way to be involved in college, **building a network for a future career,** and even awake interest in another fields. Also better support from peers that are interested in the same field. Better opportunities overall.
**elevator pitch** (8% of responses)	It would **have given me a greater understanding of how small talks with relevant people in the my desired field of study and work are important for any job position** I was interested in seeking.	If I would have gotten this assignment earlier, **I think I would have learned how to network better.** This exercise helped me hone in on what can be said, especially when speaking to a stranger that could potentially help me in future plans.	**Would have helped me network better earlier on** and continue relationships throughout my senior year.
**reference letter** (12% of responses)	This assignment would have inspired me to keep in touch with my professors and create long lasting relationships. **I would have made myself known and maintained in contact every now and then with those who could potentially write me a recommendation letter.** I would be more than a number in the class.	This would be better earlier on so that **I could be reminded to build a relationship with my professors more** to allow a better letter to be written for me.	I think that if I would have done the recommendation letter activity earlier like in my sophomore year **I would be more prepared and built stronger relationships with professors** so I would be able to get better letter of recommendations.

### Additional inductive codes connecting to “how would having the [module] during your chosen time have better prepared you for life after FIU? What would you have done differently in regards to career preparation?”

A complete list of additional inductive codes found in these data are shown in Appendix 5 in S1 File. Overall, we see connections to having more professional documents (*e*.*g*. resume and personal statement) and connections to logistics (*e*.*g*. how to document experiences, how to promote yourself, and how to ask for a reference letter). These data are relevant when implementing modules and provide insight into additional benefits of the modules for students.

### A working model for how and why our CDC influences student science and biology identities

**Fig 5:**
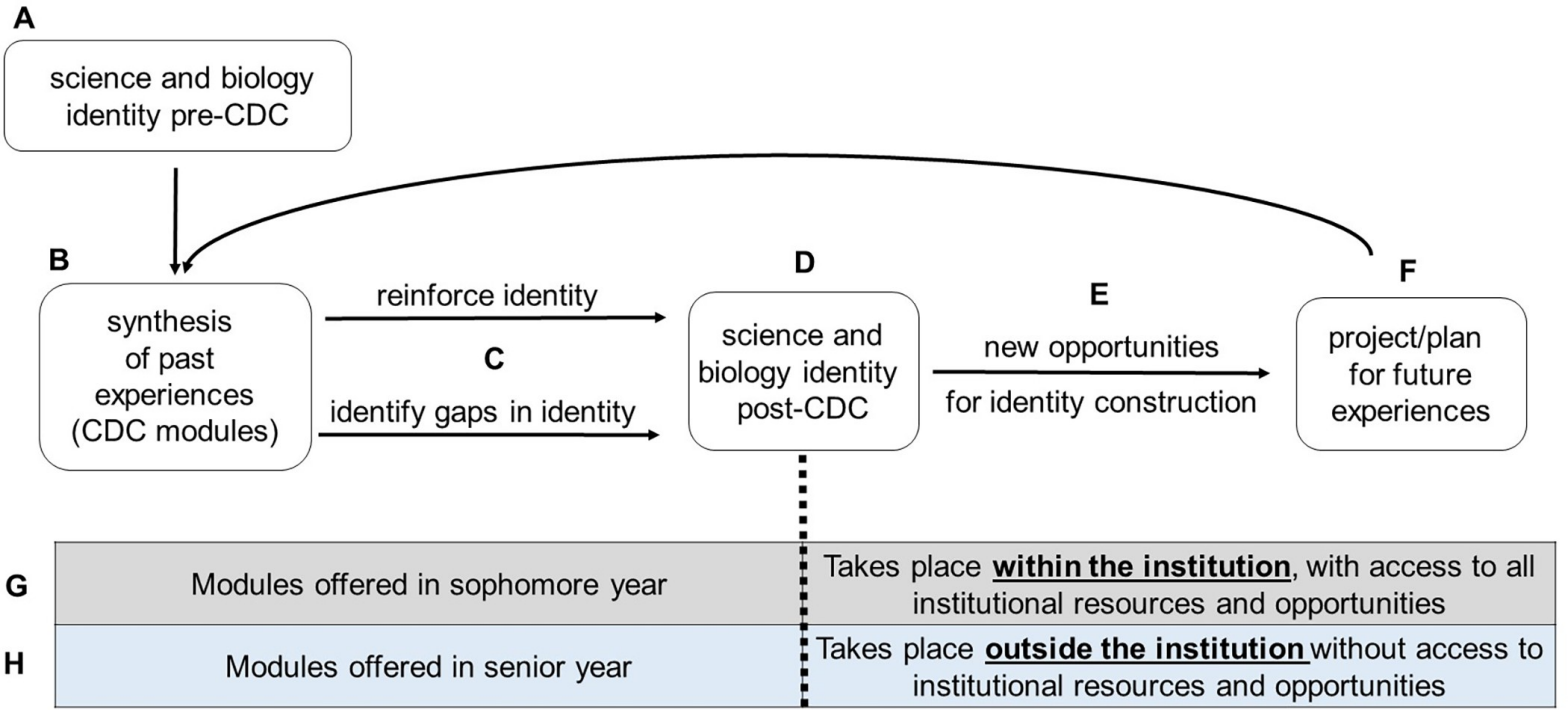
A working theoretical model summarizing our data.

Collectively, our data show that the main benefit of our modules is enabling students to synthesize their previous knowledge, skills, and experiences in biology into a more general, overarching collective experience that they are now able to place into a new context. This becomes the foundation of our working model (Fig 5). As students enter the CDC (Fig 5A) and engage with the modules, students are able to synthesize past experiences (Fig 5B). This results in two paths forward, either the reinforcement of identity or the identification of gaps in identity (Fig 5C). First, as happened for the majority of students in our study, identity is positively reinforced (Figs 1 and 2; Table 3). Second, and happening to a minority of our students, is that science identity becomes compromised (Fig 2 and Table 5). Students emerge from the CDC with a shift in identity (Fig 5D) and possible new ambitions to keep gaining experience that is relevant to their chosen career path (Fig 5E). We see evidence that engagement with the modules inspires students to identify additional skills and experiences which will further impact their identities (Fig 4 and Table 4). Participating in these new opportunities, *e*.*g*. hands-on experiences, joining biology-focused organizations, or networking opportunities, help students better project/plan for future short-term experiences that will help them advance towards their career goals (Fig 5F). Our model cycles, as students continue to engage with new experiences they have the opportunity to synthesize these new experience with past experiences (Fig 5F cycles back to Fig 5B). This agrees with science identity being dynamic, with student trajectories shifting directions over time depending on students’ lived experiences and social interactions with others [30–33].

Implementing career development early, as suggested by our students, allows for new opportunities for identity construction and planning for future experiences to take place *while students are still part of their undergraduate institutions and have access to career development resources* (Fig 5G). In this scenario, students can find new opportunities for identity construction while they are still connected to their institution and all the resources contained within. Waiting to provide career development until students are on the cusp of graduation (Fig 5H) leaves students to complete identity construction on their own and likely outside of their undergraduate institutions.

## Discussion

It is critical for biology educators to better understand the underlying potential of biology-centered CDCs. In this study, we were guided by two research questions. First, what are the mechanisms underlying the shift in student’s science and biology identities while participating in a biology-specific CDC and second, what is the optimal timing for a biology-specific CDC? To address these questions, we analyzed students’ perceptions of and experiences with the CDC through an identity lens. Our data revealed that the modules allow students to organize individual courses and experiences into a more holistic framing of their undergraduate years. Additionally, our data strongly suggests that career development should be offered earlier, and perhaps even continuously, in students’ undergraduate careers. In what follows, we contextualize key findings within our identity frameworks.

### The dangers of compartmentalization

Biology is an extremely diverse discipline, with a wide-ranging set of subdisciplines examining the life sciences from the cellular level to the ecosystem level and every level in between. Therefore, faculty members who teach biology approach the discipline from different angles and those teaching upper-level courses are often unaware of what their colleagues are teaching in introductory courses, allowing courses and curricula to become disconnected from one another [52]. This often leads to biology majors compartmentalizing their learning, reinforcing the claim that biology courses focus mostly on the recall and comprehension of facts [53]. As a result, biology majors struggle with integrating their vast content knowledge and experiences into a larger view of biology [52].

We see this same scenario play out in our data with respect to students being able to see themselves as scientists and biologists. Student responses often include statements referencing now being able to remember and/or organize their previous biology knowledge and experiences and, most importantly, the skills they have learned. Many student responses, across several different career modules, reference now being able to place their biological knowledge and experiences into a new context, *e*.*g*. as a scientist (performance/competence), as a member of the scientific community (sense of belonging), and as a future scientific professional (recognition). These data suggest that, prior to engaging with our modules, our student population seemed unaware of how to connect their experience as biology majors to being a scientist, to being a member of the scientific community, and/or to being a scientific professional. In this way, our study acts as another cautionary warning for continuing to allow students to compartmentalize their biology knowledge and skills on a class-by class basis. Not only does this system promote students lacking the ability to integrate their content knowledge into a larger view of biology, it also impedes their ability to place the totality of their undergraduate experience, and especially their biological skill set, into the context of being a future member of the scientific workforce.

### Career development should happen early and often

Our student population was in their final semester of a biology program, which is hardly the ideal timing for career development. We know our modules need to be implemented earlier in the curriculum, but we do not know what timing or what sequence would be most valuable to our students or how different timing would influence student identity. Because we are interested in building students biology identities, and since disciplinary identities can be fluid depending on context [30,45–47], understanding student’s perceptions of when modules should be implemented and what benefits modules provide is critical.

In general, students would prefer to have the majority of the modules take place during the sophomore year, which is fairly early in the curriculum (Fig 3). A sophomore year CDC is also supported by previous research in biology and the geosciences [16,44]. Interestingly, the four modules that are preferred the earliest (scientific societies, resume, skills charts, and portfolios) are also the modules with the most connections to science and biology identity (Figs 1 and 2). Recommendation letters and personal statements, which were preferred after the start of the junior year, are modules related to presenting yourself (Appendix 1 in S1 File). This suggests that students have somewhat “self-organized” themselves to spend their sophomore year learning how to apply the scientific skills and experiences of their undergraduate education to new contexts before they set about presenting themselves through personal statements and reference letters in their junior year.

We see one exception to this trend with the resume module, which was placed in the middle of the sophomore year. This is likely the result of a large percentage of our student population holding jobs in addition to being students, and needing a resume to apply for these jobs, as noted in the responses shown in the “Being better prepared” results section.

We were pleased to see that students almost uniformly spaced out the modules across the sophomore year and slightly into the junior year. We consider these data to support what we consider the ideal implementation of career development modules: implement early and implement often. There is precedent for this in the form of a “Cornerstone” course implemented in sociology. The Cornerstone course is a lower level course designed to prepare students for advanced coursework in sociology and/or assist them in understanding the nature of the major and associated career options [54]. The career development content contained within the Cornerstone course were noted in evaluations by over 70% of the students, with one student noting that she now feels confident in explaining what she can do with a degree in sociology. We offer this as a challenge to the scientific community: how can we implement an early and continuous career development, as a *Cornerstone* course instead of a *Capstone* course, to students who already have a full and demanding course load?

Meeting this challenge would have a direct effect on how students structure their undergraduate experiences, as shown in our data (Fig 4). The three modules that students asked for early, scientific societies, skills charts, and portfolio (Fig 3), are also the modules that 1) prepare students to place their knowledge and experiences into new contexts and 2) impact how students would have structured their undergraduate experience had then engaged with these modules earlier (Fig 4). In addition, students report that having six of the seven modules earlier in the curriculum would have better prepared them to apply to graduate/professional school and/or jobs (Fig 4). If students felt more prepared earlier in their undergraduate careers, would this positively affect their performance/competence in biology? Would they feel an increased recognition as biologists? Would we see a decrease in the “made me feel less like a scientist” responses we see in Table 4? Could this potential increase in identity linked to career development impact the large number of students pursuing pre-health careers who will ultimately have to adjust their career goals?

Finally, earlier implementation of the career modules may result in the interest construct of biology identity playing a larger role. With our population of graduating seniors, we see no evidence of the interest factor, either quantitatively or qualitatively [36]. This is in line with previous studies showing that the beginning and end of an undergraduate program exist in different contexts because the nature of the community around students differs. When students are surrounded by others with similar science intentions, *e*.*g*. students who have progressed to upper division science courses with other students who may also see themselves strongly as the kinds of people who engage in that science, the process of disciplinary identity construction is likely to change [31]. Specifically, first year and senior female students in undergraduate physics majors exhibit different models of identity development. While performance/competence and recognition were found to have a direct effect on physics identity for both first year and senior female undergraduates in physics, interest had an indirect effect and a newly added construct of sense of belonging had a direct effect on physics identity only for senior female physics undergraduates [31]. Therefore, implementing these modules earlier, before students reach upper division courses, may allow for the interest construct to play more of a role in building science and biology identity.

### The role of sense of belonging

Because disciplinary identity is fluid, it is also likely that with different contexts new constructs will emerge as important [31]. For our study, we see the emergence of sense of belonging. We use Strayhorn’s definition of sense of belonging as a lens through which to view our data. Specifically, “sense of belonging refers to students’ perceived social support, a feeling or sensation of connectedness, and the experience of mattering or feeling cared about, accepted, respected, valued by, and important to the community or others such as faculty, staff, and peers” [55]. We see many connections to this definition in our data. Deductive coding using science identity as a framework suggests that certain modules helped students to feel connected to the community of scientists and to the field of science (Fig 1). Data shown in Fig 2 suggests that the reference module allows students to see themselves through the eyes of their professors, helping students see how professors respect and value them. These results are in line with a previous study showing that sense of belonging was only a significant factor in identity development only for senior year students [31].

### Historically marginalized students and career development

Our study takes place at a Hispanic Serving Institution and our student demographics also show a majority of women and ~23% first generation students (Table 1). Therefore, results from this study can help to address the research gap on STEM-focused CDCs and historically marginalized students, including for first generation students [56]. While we are unable to connect student open-ended responses back to specific students, collectively our data represent a population that is 82% Hispanic (Table 1). This is important, as science identity among historically marginalized groups is especially critical and has been shown to be related to students’ persistence in a STEM major and intention to pursue a STEM career, including pursuing graduate school [19,57–59]. In addition, historically marginalized populations continue to be underrepresented in the STEM workforce [60]. Our study is a qualitative contribution to further expand research on science identity and historically marginalized populations.

### Future directions

This study provides qualitative support to quantitative data. However, we still do not have any longitudinal data on how participation in our CDC impacts students post-graduation, and longitudinal follow-ups for participants in our study would be extremely valuable. In line with most research on STEM-centered CDCs, we have shown the immediate impact on students, but we have no data on how these students retain and/or implement what they learned through the CDC once they graduate. Ultimately, the impact CDCs have on students quality of work and life after graduation remains unknown [8].

We challenge the scientific community to design novel methods for implementing an early and continuous career development sequence for biology majors and to think about early implementation through a Cornerstone lens. One possibility is the use of “pocket modules” that could be implemented into other courses throughout the biology curriculum. For example, the scientific society module can be completed in ~30 minutes and could be implemented into large, sophomore-level courses on the first day of class after the syllabus review or in a class period falling before the Thanksgiving holiday as an efficient use of class time that might otherwise go unused. Pocket modules would serve several purposes. First, they would allow for implementation of the modules earlier in the curriculum without requiring an additional course or seminar. Second, implementation across several courses would ensure large number of students having access to the modules. Third, data collection at earlier time points would shed light on whether constructs of identity development as it relates to careers changes over the course of a student’s academic career, specifically the interest and sense of belonging constructs. Fourth, data collected from students who may have engaged with a module more than once as part of the “continuous” implementation could shed light on the role reinforcement plays in career development. It is possible that having these modules spread out over several courses would lead to a completely different identity development than having the modules back-to-back in a single course.

When selecting which module to use as a pocket module, one should consider the relative value of the different modules. If only one or two modules can be implemented, we recommend considering scientific societies and skills charts for three reasons. First, qualitative data from these two modules strongly suggest that early implementation would set students on a better course for using their undergraduate years to learn more about non-medical career options, become more involved in career-related opportunities, and, most importantly, build better networks. Second, the skills charts module provides the dual benefits of 1) compelling students to explore various careers across biology and 2) helping students identifying skills they still need to develop, as well as a plan to develop these skills, during their undergraduate education. Third, the scientific societies module has the largest connections to sense of belonging, which has been shown to influence many other factors, including student persistence and retention [55]. In this sense, the scientific societies and skills charts modules serve as the Cornerstone within the Cornerstone and have strong potential for influencing the overall trajectory of student career development.

We also see benefits of the CDC that are outside of identity development, suggesting that our data can be analyzed through additional frameworks (Appendices 4 and 5 in S1 File). One possible framework for additional analysis is agency, which, in general, is defined as strategic and intentional views or actions toward goals that matter to the student [61]. Agency frameworks can be used to better understand how undergraduates navigate their career goals when the onus is on the student to advance. We see this especially with the data in Appendix 5 in S1 File, where students provide responses connected to promoting themselves, making sure their professional documents are complete and up to date, and asking for reference letters. In our work, agency and identity are likely interconnected and additional research could explore this relationship.

### Limitations

The data we collected was from a cohort of senior students who we asked to predict what would have happened if they engaged with the modules earlier. Longitudinal data from the same individuals over time would be ideal for studying the changing nature of biology identity construction relating to career development.

## Supporting information

S1 File Appendix 1. Additional data and information related to the manuscript. (DOCX)

S2 File Appendix R. 2aw data related to identity. (XLSX)

S3 File Appendix 3. Raw data related to timing of career development modules. (XLSX)
