# Supplementary material for: Cornerstone over Capstone: The case for structured career development opportunities early in the undergraduate biology curriculum as a way to influence science and biology identities
Source: PLoS One. 2023 May 5;18(5):e0285176. doi: 10.1371/journal.pone.0285176 (PMC10162542; doi:10.1371/journal.pone.0285176)

**Appendix 1.** An overview of the career development modules implemented in this study (McCartney et al., 2022).

| **Module** | **Task** | **Disciplinary Identity** |
| --- | --- | --- |
| scientific portfolios | students assemble a tangible, visual representation of their past accomplishments | performance/competence in Biology |
| scientific societies | students identify three societies that match their interests and join them | performance/competence in Biology recognition from others as a Biologist |
| skills charts | students work on matching their current skills to employer needs listed in job ads | performance/competence in Biology |
| resume workshop | students develop a professional resume | performance/competence in Biology |
| elevator pitch | students learn to present themselves professionally in a short amount of time | performance/competence in Biology  recognition from others as a Biologist |
| personal statement | students reflect and write a statement that represents their goals and visions | performance/competence in Biology |
| reference letter | students practice writing a recommendation letter for themselves | recognition from others as a Biologist |

**Appendix 2.** A detailed overview of qualitative data collection.

| Fall 2020: “please explain why the [module] did, or did not, make you feel more like a scientist.” | | |
| --- | --- | --- |
| **module** | **# of responses** | **average length of response** |
| portfolio | 117 | 29 words |
| scientific societies | 117 | 21 words |
| skills charts | 117 | 25 words |
| resume | 115 | 23 words |
| elevator pitch | 118 | 22 words |
| personal statement | 118 | 21 words |
| reference letter | 117 | 23 words |

| Spring 2021: “How would having the [module] during your chosen time have better prepared you for life after FIU? What would you have done differently in regards to career preparation?” | | |
| --- | --- | --- |
| **module** | **# of responses** | **average length of response** |
| portfolio | 99 | 36 words |
| scientific societies | 97 | 30 words |
| skills charts | 98 | 27 words |
| resume | 98 | 29 words |
| elevator pitch | 97 | 23 words |
| personal statement | 98 | 26 words |
| reference letter | 97 | 25 words |

**Appendix 3.** Science identity and Biology items used in quantitative data collection (McCartney et al., 2022) and as frameworks for interpreting qualitative data in this study.

| **Science identity (Estrada *et al.,* 2011)** | |
| --- | --- |
| I have a strong sense of belonging to the community of scientists. |  |
| I derive great personal satisfaction from working on a team that is doing important research. |  |
| I have come to think of myself as a scientist. |  |
| I feel like I belong in the field of science. |  |
| The daily work of a scientist is appealing to me. |  |
| **Biology identity (adapted from Godwin *et al.* 2016)** | |
| I am interested in learning more about biology. | Biology  interest |
| I enjoy learning biology. |  |
| My parents/relatives/friends see me as a biology person. | Biology  recognition |
| My biology teacher sees me as a biology person. |  |
| I am confident that I can understand biology in class. | Biology  performance/  competence |
| I am confident that I can understand biology outside of class. |  |
| I can do well on exams in biology. |  |
| I understand concepts I have studied in biology. |  |
| Others ask me for help in biology. |  |
| I can overcome setbacks in biology. |  |

**Appendix 4.** Additional themes found in the qualitative data in how students perceived each of the career development modules. These themes did not directly relate to Biology identity frameworks.


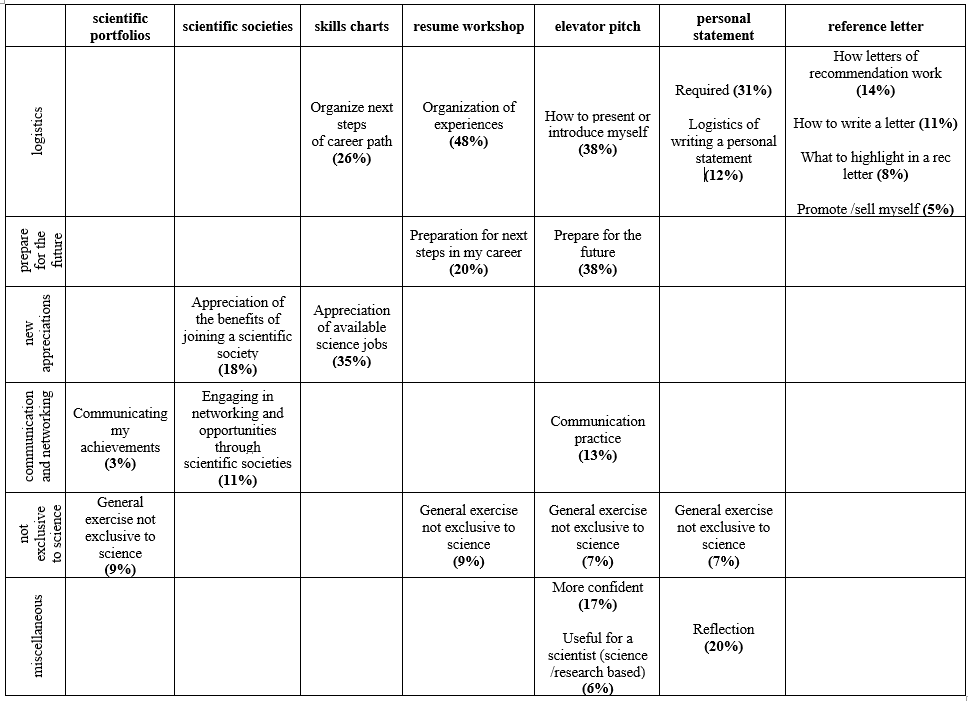


**Appendix 5.** Additional themes found in the qualitative data from the open-ended question “How would having this assignment/information during your chosen time have better prepared you for life after FIU?  What would you have done differently in regards to career preparation?”


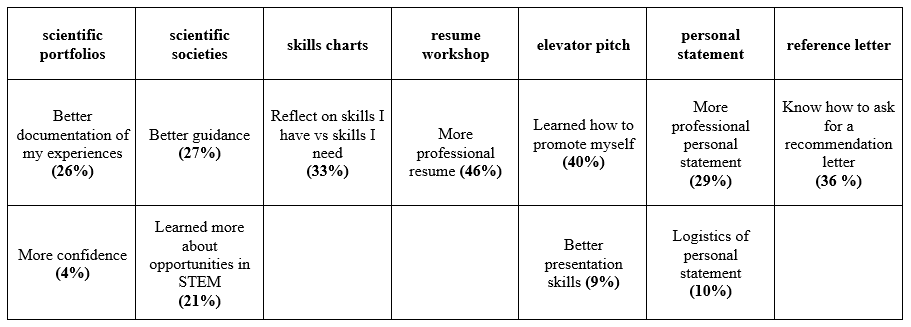

Supplement: S1 File — (DOCX) [file pone.0285176.s001.docx]
